# The Extent of Inflammatory Cell Infiltrate and Fibrosis in Lungs of Telomere- and Surfactant-Related Familial Pulmonary Fibrosis

**DOI:** 10.3389/fmed.2021.736485

**Published:** 2021-09-24

**Authors:** Aernoud A. van Batenburg, Matthijs F. M. van Oosterhout, Sebastiaan N. Knoppert, Karin M. Kazemier, Joanne J. van der Vis, Jan C. Grutters, Roel Goldschmeding, Coline H. M. van Moorsel

**Affiliations:** ^1^Department of Pulmonology, St Antonius ILD Center of Excellence, St Antonius Hospital, Nieuwegein, Netherlands; ^2^Department of Pathology, DNA Pathology, St Antonius ILD Center of Excellence, St Antonius Hospital, Nieuwegein, Netherlands; ^3^Department of Pathology, St Antonius ILD Center of Excellence, St Antonius Hospital, Nieuwegein, Netherlands; ^4^Department of Pathology, University Medical Center Utrecht, Utrecht, Netherlands; ^5^Center of Translational Immunology, University Medical Center Utrecht, Utrecht, Netherlands; ^6^Division of Heart and Lungs, University Medical Center Utrecht, Utrecht, Netherlands; ^7^Department of Clinical Chemistry, St Antonius ILD Center of Excellence, St Antonius Hospital, Nieuwegein, Netherlands

**Keywords:** inflammation, fibrosis, fibroblast foci, telomere mutations, surfactant mutations, idiopathic pulmonary fibrosis, familial pulmonary fibrosis (FPF)

## Abstract

Familial pulmonary fibrosis (FPF) is a monogenic disease most commonly involving telomere- (*TERT*) or surfactant- (*SFTP*) related mutations. These mutations have been shown to alter lymphocytic inflammatory responses, and FPF biopsies with histological lymphocytic infiltrates have been reported. Recently, a model of a surfactant mutation in mice showed that the disease initially started with an inflammatory response followed by fibrogenesis. Since inflammation and fibrogenesis are targeted by different drugs, we investigated whether the degree of these two features co-localize or occur independently in different entities of FPF, and whether they influence survival. We quantified the number of lymphocyte aggregates per surface area, the extent of diffuse lymphocyte cell infiltrate, the number of fibroblast foci per surface area, and the percentage of fibrotic lung surface area in digitally scanned hematoxylin and eosin (H&E) sections of diagnostic surgical biopsies of patients with *TERT-*related FPF (TERT-PF; *n* = 17), *SFTP*-related FPF (SFTP-PF; *n* = 7), and sporadic idiopathic pulmonary fibrosis (sIPF; *n* = 10). For comparison, we included biopsies of patients with cellular non-specific interstitial pneumonia (cNSIP; *n* = 10), an inflammatory interstitial lung disease with high lymphocyte influx and usually responsive to immunosuppressive therapy. The degree of inflammatory cell infiltrate and fibrosis in TERT-PF and SFTP-PF was not significantly different from that in sIPF. In comparison with cNSIP, the extent of lymphocyte infiltrates was significantly lower in sIPF and TERT-PF, but not in SFTP-PF. However, in contrast with cNSIP, in sIPF, TERT-PF, and SFTP-PF, diffuse lymphocyte cell infiltrates were predominantly present and lymphocyte aggregates were only present in fibrotic areas (p < 0.0001). Furthermore, fibroblast foci and percentage of fibrotic lung surface were associated with survival (*p* = 0.022 and *p* = 0.018, respectively), while this association was not observed for lymphocyte aggregates or diffuse lymphocytic infiltration. Inflammatory cells in diagnostic lung biopsies of TERT-PF, SFTP-PF, and sIPF were largely confined to fibrotic areas. However, based on inflammation and fibrosis, no differences were found between FPF and sIPF, substantiating the histological similarities between monogenic familial and sporadic disease. Furthermore, the degree of fibrosis, rather than inflammation, correlates with survival, supporting that fibrogenesis is the key feature for therapeutic targeting of FPF.

## Introduction

Familial pulmonary fibrosis (FPF) is a devastating interstitial lung disease (ILD) characterized by progressive scarring of lung parenchyma, most commonly manifesting as idiopathic pulmonary fibrosis (IPF) (1).

Current international guidelines state that although FPF develops at an earlier age (2) and the high-resolution computed tomography (HRCT) might differ (3), FPF and sporadic idiopathic pulmonary fibrosis (sIPF) are clinically and histologically indistinguishable (1, 4). However, the studies upon which this statement is based compared sIPF with the familial disease of unknown genetic etiology. Since then, it was discovered that nearly half of the FPF patients harbored disease-causing mutations (5). The mutated genes discovered in FPF can be subdivided into genes involved in telomere maintenance, most commonly in the telomerase reverse transcriptase (*TERT*) gene, and in genes involved in surfactant protein C, A1, and A2 (*SFTPC, SFTPA1*, and *SFTPA2*) (6–12). While telomere dysfunction is a systemic disease that can cause immunodeficiency (13, 14), for example, common variable immunodeficiency disease (15, 16), genetic variations in surfactant genes were also associated with changes in immune cell activation and severity of infection (17). Moreover, lungs of patients with *SFTP* mutations have been frequently described to contain lymphocytic infiltrates and were sometimes classified as desquamative interstitial pneumonia, organizing pneumonia, or cellular non-specific interstitial pneumonia (cNSIP) (12, 18–20). cNSIP is an inflammatory interstitial lung disease characterized by a high lymphocyte influx in the lungs and a diffuse fibrotic component (21). Furthermore, a genetic surfactant protein C deficiency was reported to be associated with pulmonary infections in childhood and immunosuppressive treatment is often beneficial in these patients (22), as well as in adults with cNSIP (21). Studies in *SFTP*-knockout mice and alveolar epithelial cell models showed that surfactant protein is important in inflammatory activation and regulation (23, 24). Interestingly, a recent study in a mouse model with heterozygous knockin of the known human SFTPC I73T mutation showed that disease initially started with an inflammatory response [marked by increased numbers of macrophages, neutrophils, eosinophils, and lymphocytes in bronchoalveolar lavage (BAL) fluid] followed by fibrogenesis, suggesting that immunosuppressive treatment in early disease may be beneficial (25). Furthermore, inflammation was shown to be associated with disease progression (26–28). Altogether, these findings suggest an important inflammatory component in the monogenic inflammatory disease that may be responsive to immunosuppressive drugs. However, immunosuppressive therapies were described to be harmful in patients with sIPF (29, 30), while anti-fibrotic therapy was proven to beneficially reduce the decline in forced vital capacity (FVC) in sIPF and other progressive fibrotic ILD (31–34). Therefore, caution is advised and more knowledge on the subject is urgently needed.

A characteristic of IPF lungs is the presence of temporal and spatial heterogeneous fibrosis with fibroblast foci (FF). FF are defined as clusters of matrix-depositing myofibroblasts, located at discrete sites of lung injury. They are considered a marker for the level of fibrosis in IPF lungs (35), and may also relate to the activity of the fibrotic process, since the presence of FF has been associated with the prognosis of IPF (26, 27, 36, 37), although not consistently so (38). Furthermore, by definition lymphocytes are predominantly located in dense fibrotic areas of IPF lungs, rather than in non-fibrotic areas, and are considered to be reactive to the fibrosis.

In this study, we quantified the extent and localization of diffuse lymphocyte cell infiltrate and lymphocyte aggregates as well as the number of fibroblast foci and percentage of fibrotic lung surface in diagnostic biopsies of adult patients with monogenic *TERT*- and *SFTP*-associated FPF. These data were compared with data from lung biopsies of patients with sIPF and cNSIP. Furthermore, we investigated whether these parameters were related with survival.

## Materials and Methods

### Patient and Tissue Selection

Diagnostic lung biopsies of 17 FPF patients with a *TERT* mutation (TERT-PF), 7 FPF patients with an *SFTP* (4 *SFTPC* and 3 *SFTPA2)* mutation (SFTP-PF), 10 patients with sIPF, and 10 patients with cNSIP were included in this study. FPF was determined when two or more first-degree family members also presented with pulmonary fibrosis. Furthermore, in the sIPF group, familial subjects were excluded and patients were screened negative for mutations in *TERT, TERC*, surfactant protein C (*SFTPC*), surfactant protein A2 (*SFTPA2*) exon 6, and TRF1-interacting nuclear factor 2 (*TINF2*) exon 6. Upper and lower lobe specimens were both included in the comparison analysis. For the survival analysis, upper and lower lobe data were averaged. Specimens were reviewed by an experienced lung pathologist (MFMvO) and diagnoses were in accordance with the ATS/ERS/JRS/ALAT guidelines (1).

### Digital Quantification of Inflammatory Cell Infiltrate and Fibrosis in Diagnostic Lung Biopsies

**Figure 1:**
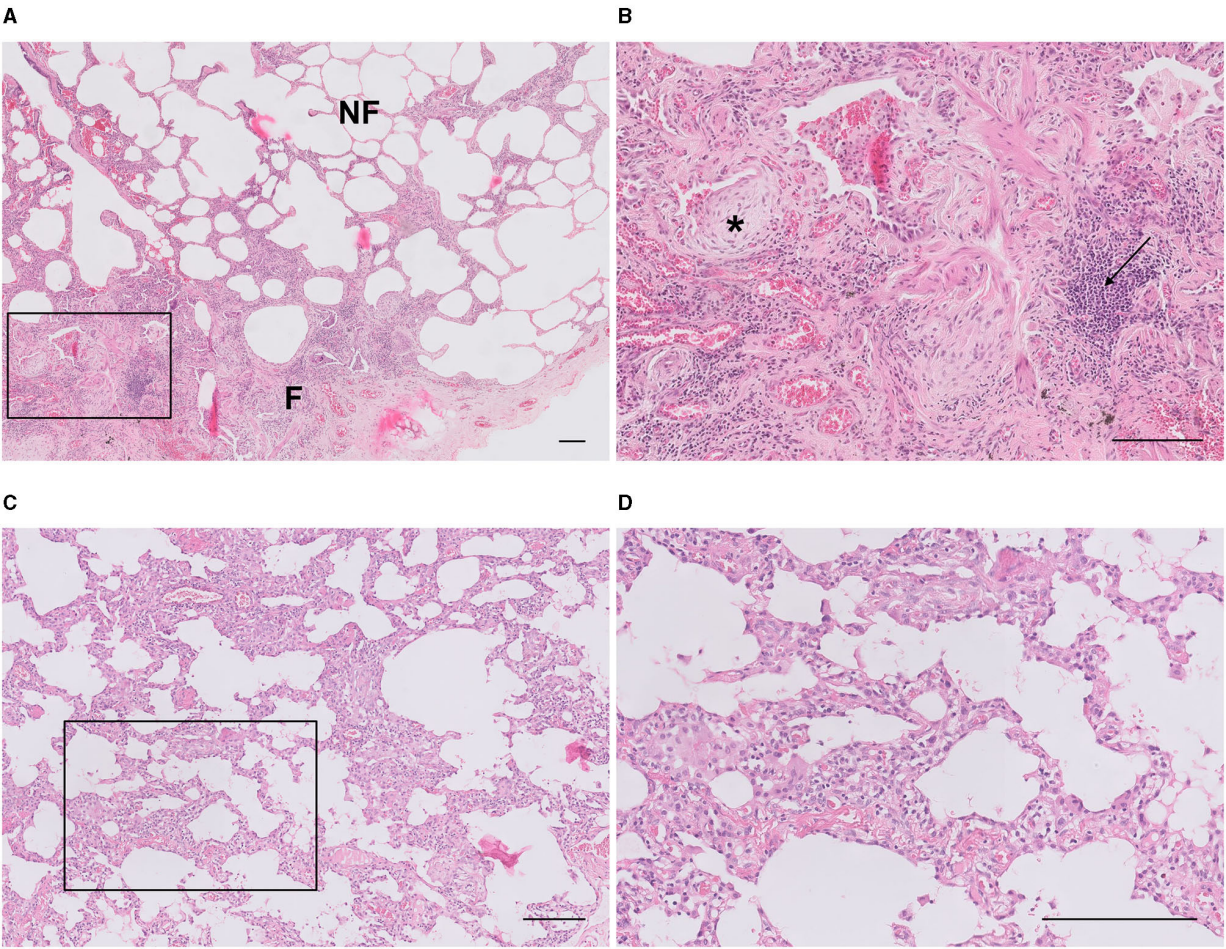
Representative Hematoxylin and Eosin (H&E)-stained diagnostic biopsies of FPF/IPF and cNSIP lungs. Images of typical **(A)** FPF/IPF and **(C)** cNSIP lung biopsies. **(B,D)** Magnifications of boxed areas in images **(A,C)**, respectively. Fibroblast foci (asterisk) and lymphocyte aggregates (arrow) were digitally quantified in the whole biopsy and the amount of diffuse inflammatory cell infiltrate (blue-purple nuclei) of the whole biopsy was scored using a histological grade from 1 to 4. Horizontal bars represent a size of 200 μm. FPF, Familial pulmonary fibrosis; IPF, idiopathic pulmonary fibrosis; cNSIP, cellular non-specific interstitial pneumonia; NF, non-fibrotic area; F, fibrotic area.

In this study, hematoxylin and eosin (H&E)-stained tissue sections were used (Figure 1). Sections were scanned for digital images at x20 magnification using ultrafast scanner 1.8 (Philips, The Netherlands) and analyzed at x5 magnification using an image management system (IMS) web application (version 3.2, Philips, The Netherlands) on a calibrated Barco display. All parameters were assessed by two independent pathologists (SNK and MFMvO). In the sections, fibroblast foci (FF) were defined as a group of spindle-shaped myofibroblasts in a matrix of collagen, mostly present in the transition zone of fibrotic and non-fibrotic areas (35). Lymphocyte aggregates (LA) were defined as sharp-edged dense groups of at least 50 lymphocytes. The size of these LAs was irrelevant. Both FFs and LAs were counted and adjusted for the total surface area of the tissue specimen. The amount of diffuse inflammatory cell infiltrate of the whole biopsy was scored using a histological grade from 1 to 4, while in a subanalysis of non-fibrotic and fibrotic areas a percentage was estimated. The histological grades refer to a very little amount 1), a little amount 2), a moderate amount 3), and a severe amount 4) of diffuse inflammatory cell infiltrate. The percentage of fibrotic lung surface was also scored.

The digital scoring of the inflammatory cell infiltrate and fibrosis was executed by experienced (MFMvO) and resident (SNK) lung pathologists. In order to master the method and to align the scoring between the pathologists, a study set of 14 biopsies was used. The final scoring for this study was performed on the analysis set. Subanalyses of fibrotic vs. non-fibrotic areas were performed on a randomly selected subset containing five samples each of sIPF, TERT, and SFTP.

### Statistical Analysis

Statistical significances were computed using non-parametric tests in GraphPad Prism version 8 (GraphPad Software, San Diego, CA, USA). In all patient groups, no significant differences were found between upper and lower lobe specimens, for all parameters. Therefore, we combined the upper and lower lobe data for each patient group. Differences between inflammatory cell infiltrate and fibrosis were determined by Mann–Whitney tests and combined Kruskal–Wallis and Dunn's multiple comparison tests. Spearman's rank coefficient was used to calculate the correlations between the number of fibroblast foci and the percentage of fibrotic lung surface. Survival was computed using Log-rank (Mantel–Cox) testing on the total population of patients with pulmonary fibrosis (TERT-PF, SFTP-PF, and sIPF combined), and the group was then divided at the median.

## Results

### Patient Inclusion

Baseline characteristics of included patients are presented in Table 1. According to the American College of Medical Genetics and Genomics (ACMG) classification of genetic variants (39), six *SFTP* variants were labeled as pathogenic and one was labeled as a variant of uncertain significance. Furthermore, we included 17 patients with a *TERT* mutation consisting of six pathogenic variants, two likely pathogenic variants, and nine variants of uncertain significance.

**Table 1:** Baseline characteristics of study groups.

	**TERT-PF**	**SFTP-PF**	**sIPF**	**cNSIP**
N	17	7	10	10
Male/Female	14/3	3/4	9/1	4/6
Mean age at time of biopsy (SD)	58.7 (9.8)	33.6 (14.1)^*^	60.1 (1, 5)	57.8 (10.1)
Mean FVC%predicted (SD)	81 (15.6)	54.8 (14.1)	73.6 (25)	78.2 (25.5)
Mean DLCO%predicted (SD)	43.6 (2, 6)	35.4 (14.1)	47.1 (25)	48.5 (12.8)
Smoking status (CS:FS:NS:U)	0:14:3:0	0:2:5:0	1:6:2:1	1:8:1:0
Pack years (SD)	18 (15.5)	3.7 (8)	20.2 (25.1)	9 (9.5)

*TERT-PF, Patients with pulmonary fibrosis and a TERT mutation; SFTP-PF, Patients with pulmonary fibrosis and an SFTP mutation; sIPF, Sporadic idiopathic pulmonary fibrosis; cNSIP, Cellular non-specific interstitial pneumonia; FVC, Forced Vital Capacity; DLCO, Diffusing Capacity of the Lungs for Carbon Monoxide; CS, Current Smoker; FS, Former Smoker; NS, Never Smoker; U, Unknown*.

* *Mean age at the time of biopsy in the SFTP-PF group was significantly lower than in the other groups (Kruskal–Wallis multiple comparison test: p < 0.01)*.

### Inflammatory Cell Infiltrate in Familial and Sporadic Idiopathic Pulmonary Fibrosis

**Figure 2:**
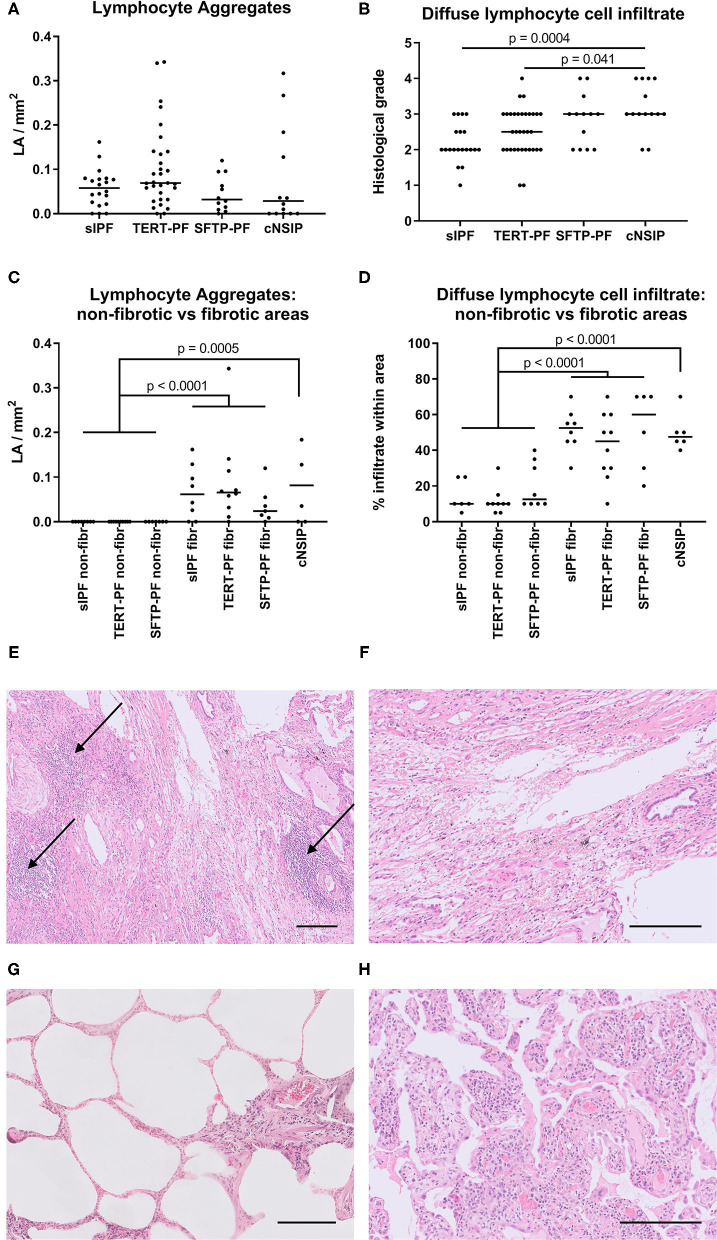
Quantification of lymphocyte aggregates and diffuse lymphocyte cell infiltrate. Scatter plots of lymphocyte aggregates (arrows in Figures 1B, 2E) and diffuse lymphocyte cell infiltrate in diagnostic biopsies of 17 TERT-PF, 7 SFTP-PF, 10 sIPF, and 10 cNSIP lungs analyzed **(A,B)** throughout the biopsy and in **(C,D)** 5 randomly selected biopsies per group to specifically assess non-fibrotic and fibrotic areas. In whole lung specimens, no significant differences were found between TERT-PF and SFTP-PF groups. In cNSIP lungs, the degree of diffuse lymphocyte cell infiltrate was significantly higher than in sIPF (*p* = 0.0004) and TERT-PF lungs (*p* = 0.04, Kruskal–Wallis test). The inflammatory cell infiltrate in non-fibrotic vs. fibrotic areas showed that lymphocyte aggregates and the diffuse lymphocyte cell infiltrate were associated with fibrotic areas in TERT-PF, SFTP-PF, and sIPF lungs (Mann–Whitney tests; *p* < 0.0001). Also the extent of lymphocyte aggregates (*p* = 0.0005) and the diffuse lymphocyte cell infiltrate (*p* < 0.0001) in cNSIP lungs was significantly higher than in non-fibrotic areas in PF lungs. No significant differences were found between fibrotic areas in PF and cNSIP lungs. Horizontal bars represent medians. **(E–H)** Examples of lymphocyte aggregates and diffuse lymphocyte cell infiltrate in Hematoxylin and Eosin (H&E)-stained diagnostic biopsies; **(E)** fibrotic area in a TERT-PF case with 3 lymphocyte aggregates (arrows) and **(F)** grade 2 diffuse lymphocyte cell infiltrate; **(G)** non-fibrotic area in an sIPF case with no lymphocyte aggregates and grade 1 diffuse lymphocyte cell infiltrate; (**H**) cNSIP case with grade 4 diffuse lymphocyte cell infiltrate. Horizontal bars represent a size of 200 μm. TERT-PF, Patients with lung fibrosis and a TERT mutation; SFTP-PF, Patients with lung fibrosis and a surfactant mutation; sIPF, Sporadic idiopathic pulmonary fibrosis; cNSIP, Cellular non-specific interstitial pneumonia; LA, Lymphocyte aggregates.

To study the degree of inflammatory cell infiltrate, we quantified the number of lymphocyte aggregates and the extent of diffuse lymphocyte cell infiltrates in TERT-PF, SFTP-PF, sIPF, and cNSIP lungs. On analyzing whole biopsies, no significant differences in lymphocyte and fibrotic parameters were found among TERT-PF, SFTP-PF, and sIPF study groups (Figures 2A,B). Comparison with cNSIP showed that in TERT-PF and sIPF, significantly less diffuse lymphocyte infiltrates were present. There was no correlation between lymphocyte aggregates and diffuse infiltrates. Detailed subanalysis of inflammatory cell infiltrates in fibrotic vs. non-fibrotic areas showed that lymphocyte aggregates were completely associated while diffuse lymphocyte cell infiltrates were strongly associated with fibrotic areas in TERT-PF, SFTP-PF, and sIPF (Mann–Whitney tests between non-fibrotic and fibrotic areas; *p* < 0.0001, Figures 2C,D). The number and extent of lymphocyte aberrations in cNSIP biopsies were comparable with that observed in the fibrotic areas of the TERT-PF, SFTP-PF, and sIPF study groups (Figures 2C–G).

### Fibrosis in Familial and Sporadic Idiopathic Pulmonary Fibrosis

**Figure 3:**
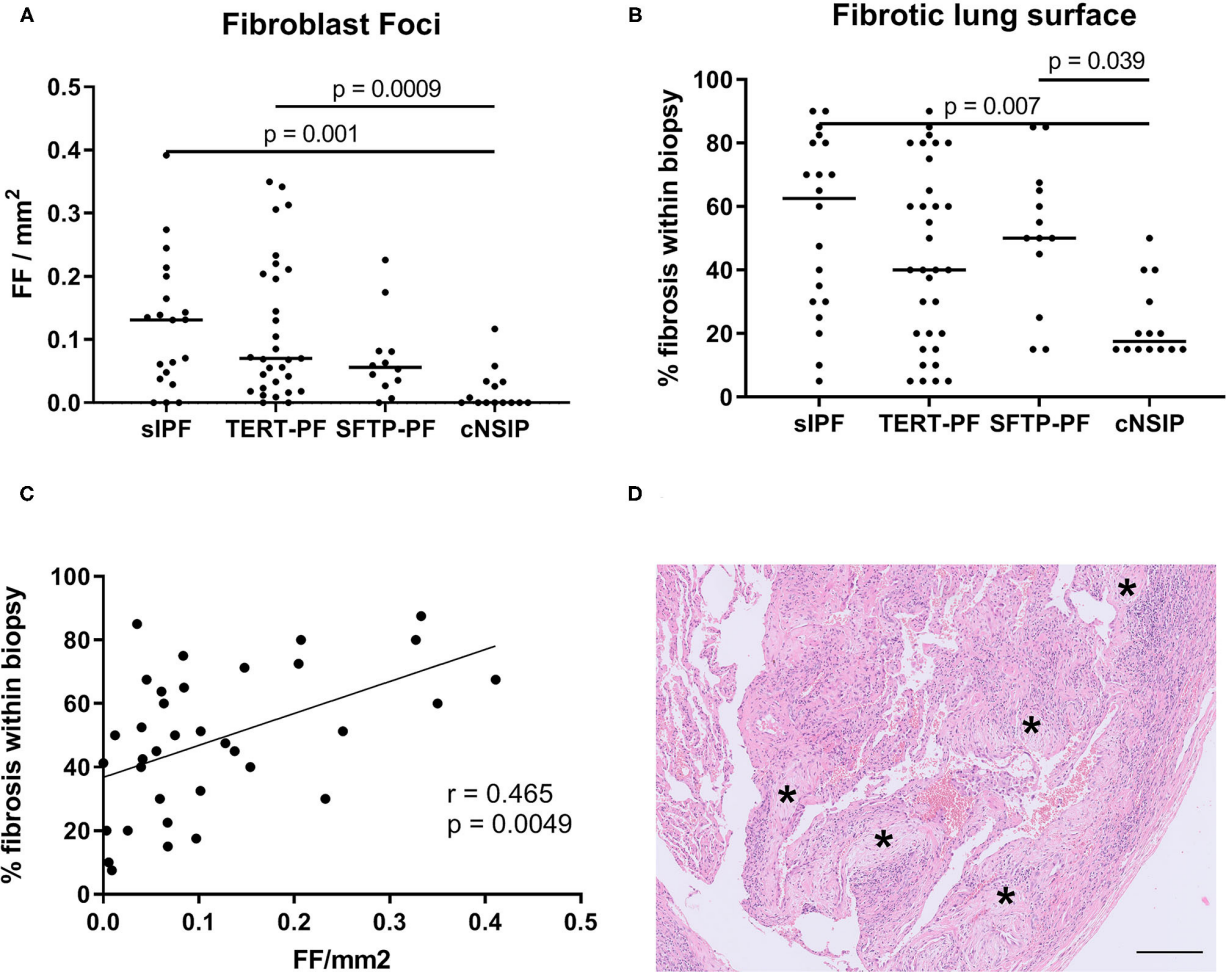
Quantification of fibrotic foci and fibrotic lung surface. Scatter plots of **(A)** the number of fibroblast foci (asterisk in Figure 1B) and **(B)** the percentage of fibrotic lung surface in diagnostic biopsies of 17 TERT-PF, 7 SFTP-PF, 10 sIPF, and 10 cNSIP lungs. For both parameters, no significant differences were found among TERT-PF, SFTP-PF, and sIPF lungs. The amount of fibroblast foci per mm^2^ in cNSIP lungs was significantly lower than in sIPF (*p* = 0.001) and TERT-PF lungs (*p* = 0.0009), while the percentage of fibrotic lung surface in cNSIP lungs was significantly lower than in sIPF (*p* = 0.007) and SFTP-PF lungs (*p* = 0.04). *P*-values were calculated using Kruskal–Wallis tests. Bars represent medians. **(C)** Positive Spearman correlation between the number of fibroblast foci and percentage fibrotic lung surface of TERT-PF, SFTP-PF, and sIPF lungs (*r* = 0.465, *p* = 0.005). **(D)** Example of a Hematoxylin and Eosin (H&E)-stained fibrotic area in an SFTP-PF lung containing 5 fibroblast foci (asterisks). Horizontal bar represents a size of 200 μm. TERT-PF, Patients with lung fibrosis and a TERT mutation; SFTP-PF, Patients with lung fibrosis and a surfactant mutation; sIPF, Sporadic idiopathic pulmonary fibrosis; cNSIP, Cellular non-specific interstitial pneumonia; FF, Fibroblast foci.

Next, we investigated the degree of fibrosis by assessing the number of fibroblast foci and the percentage of fibrotic lung surface in all four patient groups. Similar to the inflammatory cell infiltrate, no differences were found based on these fibrotic parameters among TERT-PF, SFTP-PF, and sIPF lungs (Figures 3A,B,D). Fibroblast foci in cNSIP lungs were almost absent and significantly lower than in sIPF lungs (*p* = 0.001, Figure 3A) and TERT-PF lungs (*p* = 0.0009, Figure 3A). Also, the percentage of (diffusely orientated) fibrotic lung surface in cNSIP lungs was significantly lower than in sIPF lungs (*p* = 0.007, Figure 3B) and SFTP-PF lungs (*p* = 0.039, Figure 3B). Furthermore, we found a significant moderate correlation between the number of fibroblast foci and the percentage of fibrotic surface in the fibrotic lungs (*r* = 0.465, *p* = 0.005, Figure 3C).

### Elevated Numbers of Fibroblast Foci and a High Percentage of Fibrotic Lung Surface Associated With Low Survival

**Figure 4:**
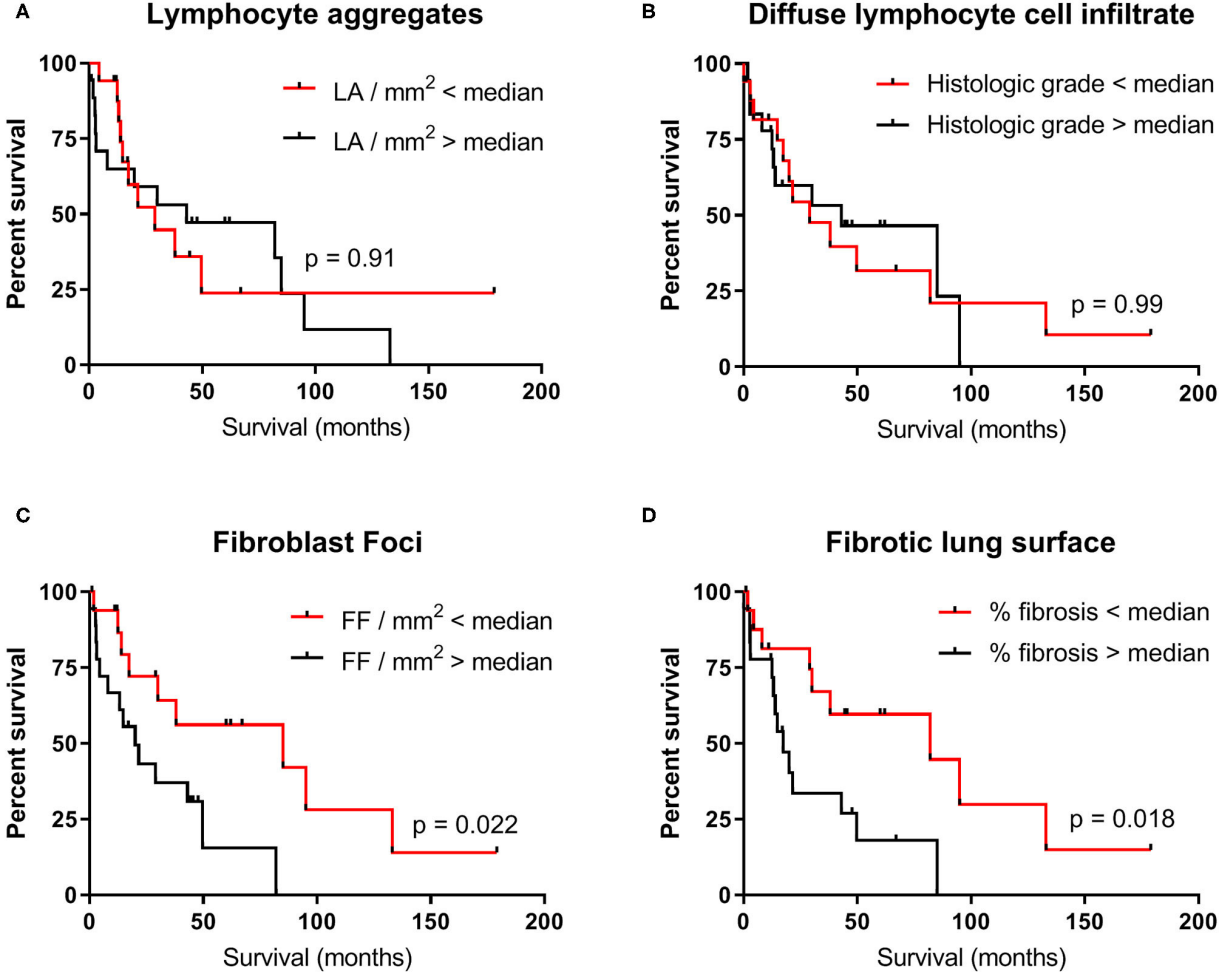
Survival of the combined TERT-PF, SFTP-PF and sIPF patient cohorts. Kaplan–Meier curves showing the association between survival in months and the degree of **(A)** lymphocyte aggregates, **(B)** diffuse lymphocyte cell infiltrate, **(C)** fibroblast foci, and **(D)** fibrotic lung surface in 34 patients with pulmonary fibrosis. Median values of each parameter were used as cut-off. For the degree of lymphocyte aggregates and diffuse lymphocyte cell infiltrate, no significant differences were found between survival above (black line) and below median (red line). For the degree of fibroblast foci and fibrotic lung surface, the significant difference in median survival was respectively 65 months (85 vs. 20 months, *p* = 0.02) and 64.6 months (82 vs. 17.4 months, *p* = 0.02). Significances were calculated using Log-rank (Mantel–Cox) tests. LA, Lymphocyte aggregates; FF, Fibroblast foci.

We investigated whether the number of fibroblast foci, percentage of fibrotic lung surface, number of lymphocyte aggregates, and extent of lymphocyte cell infiltrate were associated with survival. As there were no differences observed among TERT-PF, SFTP-PF, and sIPF groups, we combined the data. Median values of each parameter were used as cut-off. Kaplan–Meier curves showed that both elevated numbers of fibroblast foci (cut-off value: 0.075 FF/mm^2^, median survival: 85 vs. 20 months; *p* = 0.022, Figure 4C) and high percentage of fibrosis (cut-off value: 50%, median survival: 82 vs. 17 months; *p* = 0.018, Figure 4D) were significantly associated with survival. The number of lymphocyte aggregates (cut-off value: 0.062 LA/mm^2^, *p* = 0.91, Figure 4A) and the extent of diffuse lymphocyte cell infiltrate (cut-off value: 2.5, *p* = 0.99, Figure 4B) were not associated with survival.

## Discussion

In this study, we quantified the levels of inflammatory cell infiltrate and fibrosis in FPF with a *TERT* or *SFTP* mutation, sIPF, and cNSIP lungs. The degree of inflammatory cell infiltrate and fibrosis were not different among TERT-PF, SFTP-PF, and sIPF lungs. Importantly, inflammatory cell abnormalities were almost exclusively located in the fibrotic areas of the lung, with levels similar to what we observed throughout the biopsies of cNSIP lungs, while non-fibrotic locations were histologically healthy. Furthermore, survival in patients with FPF and sIPF was associated with the number of fibroblast foci and percentage of fibrotic lung surface, but not with inflammatory cell infiltration and aggregation.

Previous reports on *SFTP*-related FPF lung biopsies frequently described lymphocyte cell infiltrate and used a classification such as desquamative interstitial pneumonia, organizing pneumonia, or cNSIP (12, 18–20). Furthermore, it was reported that in mice, induction of *SFTP*- or *TERT*-related dysfunctional alveolar epithelial cells was sufficient to cause inflammatory cell infiltrate and fibrosis (25, 40, 41). Since surfactant mutations have been associated with immunological changes (17, 24), we expected the inflammatory status to be higher in SFTP-PF lungs when compared to sIPF lungs. However, we found no differences in the grade of inflammatory cell infiltrate between monogenetic fibrotic lung disease and sIPF, thereby further supporting the histological similarities between pulmonary fibrosis of known and unknown cause.

Interestingly, a comparison of the grade of diffuse lymphocyte cell infiltrate showed no difference between SFTP-PF and cNSIP biopsies. However, while lymphocyte aggregates and diffuse lymphocyte cell infiltrate are present throughout the biopsy of cNSIP lung, they were respectively absent and nearly absent (approximately 10% of the tissue surface) in non-fibrotic areas of SFTP-PF, TERT-PF, and sIPF lungs. Previously it was shown that mice with heterozygous knockin of the known human SFTPC I73T mutation developed early inflammation and no fibrosis, while the development of fibrosis occurred in homozygous mice (25). This study suggested that an early inflammatory phase precedes the development of fibrosis in mice, and disease evolution is influenced by the number of *SFTP* mutant alleles. In human disease, it is unclear whether inflammation is a cause or consequence of fibrosis. We demonstrated that an inflammatory component was absent in the non-fibrotic areas, suggesting that inflammation does not precede fibrogenesis and may even be reactive to fibrogenesis. Furthermore, in contrast to cNSIP lungs (42, 43), we found no association between lymphocytes and survival in FPF lungs, further supporting that the degree of lymphocyte aberrations in FPF lungs may not contribute significantly to disease pathogenesis (26, 44). In contrast, it has been reported that elevated numbers of lymphocytes are associated with less progressive fibrosis in patients with sIPF (45, 46) and cause resistance to bleomycin in mouse models of pulmonary fibrosis (47).

The role of different lymphocyte subtypes in IPF lungs is currently open to discussion. Previously it has been shown that T-lymphocytes were diffusely present and lymphocyte aggregates were organized in tertiary lymphoid structures, where a core of B-lymphocytes was surrounded by T-lymphocytes (48, 49). Furthermore, even though we showed that the amount of diffuse lymphocyte infiltrate in cNSIP was significantly higher than in IPF, it was previously demonstrated that the amount of different lymphocyte subtypes in IPF lungs did not significantly differ from NSIP lungs (50). However, this does not indicate that subtyping is unimportant. It has been reported that lymphocyte aggregates in IPF consist of active non-proliferating CD40L-positive lymphocytes and mature dendritic cells (49). The presence of these particular cells in IPF lungs might explain the ineffectiveness of anti-inflammatory drugs, because they are less sensitive to these agents (51). Therefore, other drugs targeting the CD40L pathway could potentially be an anti-inflammatory target in IPF. However, although CD40L immunotherapy is used in cancer, future studies should focus on its effectiveness in IPF. Continued efforts to understand the precise role of inflammation and the possible anti-inflammatory treatment in fibrotic lungs remain warranted.

It is important to note that the degree of fibrosis in FPF biopsies was significantly higher than in cNSIP but not sIPF. Combining the PF data we found that the number of fibroblast foci and the percentage of fibrotic lung surface in PF lung were significantly associated with survival. This corresponds to previous findings in sIPF (26, 27, 36, 46) and underlines the critical role of fibrotic remodeling in the outcome of patients with FPF.

Little is known about drug effects in FPF. We recently showed in a review that in patients and cell or mouse models with a surfactant-related mutation, the outcome of drugs was highly variable and most likely mutation-specific (52). Furthermore, immunosuppressive therapies require careful consideration because of the harm caused in patients with sIPF (29, 30), further emphasizing the non-causative role of inflammatory cell infiltrate in these fibrotic lungs. We showed that the absence of lymphocytic abnormalities in non-fibrotic areas of FPF lungs, the similarities with sIPF, and the association between increased fibrosis and decreased survival support fibrogenesis and not inflammation as the primary target for therapy. Since familial disease resembles sIPF, the preferred choices of drugs are antifibrotics pirfenidone and nintedanib (31, 32). However, antifibrotics in SFTP-PF patients have not been studied yet, and a previous small retrospective study including 33 TERT-PF patients treated with pirfenidone demonstrated no beneficial effect post treatment initiation (53). These data emphasize the need for prospective FPF gene or mutation-specific therapies and registration of treatment effects of current antifibrotic drugs. However, a recent retrospective European study on the effect of antifibrotics in 89 IPF patients carrying telomere-related mutations showed that pirfenidone and nintedanib were safe and reduce the decline in FVC (54).

Strengths of this study comprise the detailed analyses of inflammatory cell infiltrate and fibrosis, including the number of lymphocyte aggregates, the extent of diffusely-orientated lymphocytes, number of fibroblast foci, and percentage of fibrotic lung surface in well characterized groups of pulmonary fibrosis. Furthermore, this is the first study to show the association between survival and the degree of fibrosis as well as the number of fibroblast foci in FPF. However, two limitations are worthy to be noted. Lymphocyte aggregates were assessed in diagnostic biopsies. However, we did not routinely differentiate between different types of lymphocytes in these aggregates and we cannot exclude the possibility that we might have accidentally included some monocytes within the infiltrate. Second, while the tissue was sufficiently available for patients with TERT-PF and sIPF, lung biopsies of adult patients with SFTP-PF were extremely rare and only seven lungs were included.

The international guidelines state that generally FPF and sIPF are histologically and clinically indistinguishable (1). However, to date, no study has compared histologic inflammatory and fibrotic features of FPF patients with a specific *TERT* or *SFTP* gene mutation and sIPF. This is the first study investigating in detail the degree of inflammatory cell infiltrate and fibrosis in diagnostic biopsies of TERT-PF, SFTP-PF, sIPF, and cNSIP. We found no differences between FPF and sIPF, thereby further supporting the histological similarities between monogenic familial pulmonary fibrosis and sIPF. Furthermore, this study showed that survival among patients with FPF and sIPF depends on the number of fibroblast foci and percentage of fibrotic lung surface, but no clinically relevant correlation with inflammatory cell infiltrate was found. This corresponds with the general failure of trials with anti-inflammatory drugs and the more promising results of therapies targeting fibrosis.

## Data Availability Statement

The raw data supporting the conclusions of this article will be made available by the authors, without undue reservation.

## Ethics Statement

The studies involving human participants were reviewed and approved by Medical Research Ethics Committees United. The patients/participants provided their written informed consent to participate in this study. Written informed consent was obtained from the individual(s) for the publication of any potentially identifiable images or data included in this article.

## Author Contributions

AB, MO, KK, JV, and CM contributed to the conception and design of the study. MO and SK performed the digital quantification of inflammatory and fibrotic markers in H&E-stained diagnostic biopsies. AB carried out the analysis. MO, JG, RG, and CM supervised the study. AB and CM wrote the initial manuscript. All authors drafted and approved the final manuscript.

## Funding

This research was enabled by ZonMW-TopZorg St Antonius Science Corner grant (grant number 842002003; www.zonmw.nl; JG, CM, JV, and AB) and St Antonius Research fund (CM).

## Conflict of Interest

The authors declare that the research was conducted in the absence of any commercial or financial relationships that could be construed as a potential conflict of interest.

## Publisher's Note

All claims expressed in this article are solely those of the authors and do not necessarily represent those of their affiliated organizations, or those of the publisher, the editors and the reviewers. Any product that may be evaluated in this article, or claim that may be made by its manufacturer, is not guaranteed or endorsed by the publisher.
